# Precocious Metamorphosis of Silkworm Larvae Infected by BmNPV in the Latter Half of the Fifth Instar

**DOI:** 10.3389/fphys.2021.650972

**Published:** 2021-05-10

**Authors:** Ping-Zhen Xu, Mei-Rong Zhang, Xue-Yang Wang, Yang-Chun Wu

**Affiliations:** ^1^Jiangsu Key Laboratory of Sericultural Biology and Biotechnology, School of Biotechnology, Jiangsu University of Science and Technology, Zhenjiang, China; ^2^Key Laboratory of Silkworm and Mulberry Genetic Improvement, Ministry of Agriculture, Sericultural Research Institute, Chinese Academy of Agricultural Sciences, Zhenjiang, China

**Keywords:** *Bombyx mori*, *Bombyx mori* nucleopolyhedrovirus, prothoracic gland, transcriptome, 20-hydroxyecdysone

## Abstract

The mulberry silkworm (*Bombyx mori*) is a model organism, and BmNPV is a typical baculovirus. Together, these organisms form a useful model to investigate host–baculovirus interactions. Prothoracic glands (PGs) are also model organs, used to investigate the regulatory effect of synthetic ecdysone on insect growth and development. In this study, day-4 fifth instar silkworm larvae were infected with BmNPV. Wandering silkworms appeared in the infected groups 12 h earlier than in the control groups, and the ecdysone titer in infected larvae was significantly higher than that of the control larvae. We then used RNA sequencing (RNA-seq) to analyze silkworm PGs 48 h after BmNPV infection. We identified 15 differentially expressed genes (DEGs) that were classified as mainly being involved in metabolic processes and pathways. All 15 DEGs were expressed in the PGs, of which *Novel01674*, *BmJing*, and *BmAryl* were specifically expressed in the PGs. The transcripts of *BmNGDN*, *BmTrypsin-1*, *BmACSS3*, and *BmJing* were significantly increased, and *BmPyd3*, *BmTitin*, *BmIGc2*, *Novel01674*, and *BmAryl* were significantly decreased from 24 to 72 h in the PGs after BmNPV infection. The changes in the transcription of these nine genes were generally consistent with the transcriptome data. The upregulation of *BmTrypsin-1* and *BmACSS3* indicate that these DEGs may be involved in the maturation process in the latter half of the fifth instar of silkworm larvae. These findings further our understanding of silkworm larval development, the interaction between BmNPV infection and the host developmental response, and host–baculovirus interactions in general.

## Introduction

The mulberry silkworm (*Bombyx mori*) has been reared for the past 5,000 years in China due to its importance for silk production. In addition to this economic importance, *B. mori* has recently played an essential role as a model organism in scientific research, molecular biology, and genetics studies (Mita et al., 2004; Xia et al., 2004). *B*. *mori* undergoes complete (egg–larva–pupa–adult) metamorphosis within each generation; however, only the larval stage feeds. In general, silkworm larvae are tetramolters that proceed through four instars, molting between each instar. The durations of the larval instar stages are as follows: 3–4 days in the first instar, 2–3 days in the second instar, 3–4 days in the third instar, 5–6 days in the fourth instar, and 6–8 days in the fifth instar. However, the duration of the larval stage depends on the silkworm strain and rearing temperature. Silkworm larvae grow rapidly, and the weight of a terminal fifth instar larva is ∼10,000 times that of a newly hatched larva (Xu et al., 2019). In particular, day 3 of the fifth instar larvae is the boundary for the larval stage, where the larvae feed and grow quickly from day 1 to day 3 of the fifth instar. The larvae then (in the gluttonous stage) greatly synthesize silk proteins in the silk gland (Xu et al., 2019), which indicates maturation and leads to spinning in the terminal fifth instar stage. After the completion of silk spinning, the silkworms proceed with larval–pupal metamorphosis.

Sericulture is one of the main sources of income for farmers in many developing countries, such as China, India, Brazil, Vietnam, and Thailand (Jiang and Xia, 2014). China produces almost 80% of cocoons worldwide. Sericulture faces biological challenges from pathogenic fungi, bacteria, and viruses, which can cause annual cocoon production losses of 20–30% (Jiang et al., 2013a). Although antibiotics are administered to silkworms to prevent and treat bacterial diseases, and fresh lime and chlorine-containing preparations are used to disinfect the rearing seat to prevent fungal diseases, there are no effective prevention and treatment methods for viral diseases. Viral diseases are responsible for almost 80% of the annual cocoon production losses, and *B*. *mori* nucleopolyhedrovirus (BmNPV) is one of the major pathogens and the most prevalent threat to sericulture in almost all countries worldwide (Jiang et al., 2013a; Jiang and Xia, 2014). BmNPV is an enveloped double-stranded DNA virus that presents a biphasic infection process throughout its viral life cycle, generating progeny with two different phenotypes, namely, occlusion-derived virus (ODV) and budded virus (BV) (Gomi et al., 1999). ODVs are packaged in occlusion bodies (OBs). Both forms play different roles during pathogenesis. The alkalinity of the silkworm midgut triggers the dissolution of OBs and the release of ODV in the midgut lumen. The ODV is responsible for the primary infection through oral transmission of the virus among silkworm larvae, while the BV is responsible for the secondary infection, causing systemic spreading all over the host within the infected silkworm larvae (Jiang, 2021).

*Bombyx mori* is a model organism, and BmNPV is a typical baculovirus (Gomi et al., 1999; Mita et al., 2004; Xia et al., 2004), and together, they present an important model to assess host–baculovirus interactions (Jiang and Xia, 2014). Insights from previous host studies revealed that innate antiviral immunity in lepidopteran insects plays important roles in host–baculovirus interactions (Jiang, 2021). Antiviral proteins, including red fluorescent proteins (RFPs) (Sunagar et al., 2011; Manjunatha et al., 2018), Bmlipase (Bmlipase-1 and Bmlipase member H-A) (Ponnuvel et al., 2003; Zhang S. Z. et al., 2020), serine proteases (SPs), and serine protease homologs (SPHs) (Nakazawa et al., 2004; Ponnuvel et al., 2012), show strong antiviral activity in the digestive juice of the silkworm. Moreover, heat shock protein 19.9 (*Bmhsp19.9*) is involved in antiviral immunity against BmNPV function (Jiang et al., 2021b). BmNPV has also evolved diverse mechanisms to counter host responses and ensure its replication. For example, BmNPV activates the expression of *BmPGRP2-2* to inhibit *phosphatase and tensin homolog* (*PTEN*), which relieves its suppression of the PI3K-Akt pathway and triggers an increase in Akt phosphorylation (p-Akt) to inhibit cell apoptosis; the resulting increased cell survival is beneficial for viral replication (Jiang et al., 2019). *BmSpry* is upstream of ERK and JNK and is downregulated by BmNPV to elevate p-ERK and ensure viral reproduction in the silkworm (Guo et al., 2019). BmNPV activates the host ERK and JNK signal pathways for efficient replication (Katsuma et al., 2007). The baculovirus ecdysteroid UDP-glucosyltransferase gene (*egt*) encoding the enzyme ecdysteroid UDP-glucosyltransferase catalyzes the transfer of glucose from UDP-glucose to ecdysteroid molting hormones, and the expression of this enzyme blocks the molting of infected larval insects (O’Reilly and Miller, 1989). The BmNPV *egt* gene prolongs the survival time of infected silkworms to increase virus reproduction (Katsuma and Shimada, 2015).

The expression of NPV genes occurs in four phases: immediate early phase (0–4 h post-infection, hpi), delayed early stage (5–7 hpi), late stage (8–18 hpi), and very late stage (>18 hpi). Viral DNA replication starts at 8 hpi and represents the transition from the early stage to the late stage (Huh and Weaver, 1990; Jiang et al., 2013b, 2021a). Global shutoff of host gene expression and protein synthesis in insect cells begins at the early stage at around 12–18 h after NPV infection (Du and Thiem, 1997; Shirata et al., 2010; Ikeda et al., 2013). However, previous studies that investigated the interactions between BmNPV and its hosts have mainly focused on newly exuviated fifth instar silkworm larvae infected by BmNPV and the systemic process of infection by BmNPV within 48 hpi (i.e., silkworm larvae in the first half of the fifth instar). Until now, no studies have investigated the interactions between BmNPV and silkworm larvae in the latter half of the fifth instar.

The use of next-generation sequencing technologies in genome-wide studies of silkworms and BmNPV interactions is a recent development and is rapidly advancing. Recently, several studies have reported on the transcriptional response of silkworm larvae against BmNPV infection in the major innate immune tissues of the fat body and midgut (Chen et al., 2019; Huang et al., 2019; Jiang et al., 2019; Toufeeq et al., 2019; Zhang X. et al., 2020). However, the gene expression of prothoracic glands (PGs) infected by BmNPV has not yet been analyzed.

In the present study, we first investigated the precocious molting and metamorphosis of silkworm larvae under BmNPV infection, and the ecdysone titer in infected larvae was significantly higher than that of the control larvae. We then used RNA sequencing (RNA-seq) to analyze silkworm PGs 48 h after BmNPV infection. The classifications of the 15 differentially expressed genes (DEGs) were mainly involved in the metabolic processes and pathways. The reverse transcription quantitative PCR (RT-qPCR) results of the DEGs in the PGs of BmNPV-infected larvae at 24, 48, and 72 h were generally consistent with the transcriptome data. The transcripts of *BmTrypsin-1* and *BmACSS3* were significantly increased from 24 to 72 h after BmNPV infection, indicating that they may be involved in the maturation process in the latter half of the fifth instar of silkworm larvae. This study was conducted to further our understanding of the complex biological processes in the interactions between BmNPV and its precocious metamorphic insect hosts.

## Materials and Methods

### Study Animals and Virus

*Bombyx mori* F_50_ strain larvae were reared on fresh mulberry leaves under a 12:12 h day/night cycle at 25 ± 1°C and 60% relative humidity. The majority of the fifth instar larvae started wandering on day 8, depending on the batch of the silkworm. The larvae underwent oral inoculation with a wild BmNPV T3 strain, and the OBs were obtained from the larvae hemolymph before the larvae died. The OBs were purified by repeated and differential centrifugation, as previously described (Rahman and Gopinathan, 2004).

### Sample Collection

In total, 500 day-4 fifth instar larvae were orally infected with BmNPV using 2.0 × 10^6^ OB/larva. Control larvae (*n* = 500) were fed the same volume of sterile distilled water. The larvae of the infected and control groups were maintained in isolation and reared under the same conditions. The PGs were entwined in pairs in the tracheal bush of the first spiracle (Supplementary Figure 1). The PGs were carefully removed from the larvae of the infected and control groups after 24, 48, and 72 h (Supplementary Figure 2). Hemolymph was collected from day 6 (48 hpi), day 6.5 (60 hpi), day 7 (72 hpi), and day 7.5 (84 hpi) fifth instar larvae of the infected and control groups for use in assays of the ecdysteroid titers among the different developmental stages.

### Statistics of Precocious Maturation of Silkworms After BmNPV Infection

Day-4 fifth instar larvae were divided into 6 groups with 200 in each group. All 200 larvae used in each of the three independent experiments were orally infected with BmNPV using 2.0 × 10^6^ OB/larva. The 200 control larvae used in each of the three independent experiments were fed with the same volume of sterile distilled water. The larvae of the infected and control groups were maintained in isolation and reared under the same conditions. Diseased and dead larvae were removed and counted during rearing. When the proportion of mature silkworms was > 5% (first gate), the statistics was started.

### Cholesterol and 7-Dehydrocholesterol Feeding Experiments

As previously described (Wu et al., 2016), silkworm larvae were fed mulberry leaves supplemented with 8,000 mg/L of cholesterol and 7-dehydrocholesterol (7dC). Mulberry leaves supplemented with the same volume of sterile distilled water were used as the control. Day-5 fifth instar larvae were initially fed (first feed session) with cholesterol and 7dC supplemented leaves and then again 24 h later (second feed session). Replacement mulberry leaves were added 6 h after each feeding session. The proportion of mature vs. immature larvae was counted, and the second gate was determined to be the point at which the majority of the larvae had started maturing. The point at which all larvae (100%) had reached maturity defined the third gate. All experiments were repeated three times per group.

### Assay of Ecdysteroid Titers in Hemolymph and Examination of Viral DNA in PGs

The hemolymph samples were homogenized in 50% MeOH (800 μl). The resultant homogenates were centrifuged, and the supernatant was used to assay the ecdysteroid titers using an Insect Ecdysone ELISA Kit (Shanghai MEILIAN Biotechnology Co., Ltd.) according to the manufacturer’s instructions. RT-PCR was used to analyze the BmNPV virus replication level. The total DNA was extracted from the PGs of the BmNPV-infected larvae at 24, 48, and 72 h, as well as from the control larvae at 48 h. The DNA templates (10 ng) were PCR amplified using primers for the BmNPV *GP41* gene. The silkworm glyceraldehyde-3-phosphate dehydrogenase (*BmGAPDH*) was used as the internal control. The specific primers for each gene used in the RT-PCR are shown in Supplementary Table 1. The RT-PCR product of each gene was defined as previously described (Zhang et al., 2019).

### Transcriptome Analysis

Total RNA was isolated from the PGs of the silkworm larvae using the TRIzol reagent (Invitrogen, New York, NY, United States) according to the manufacturer’s instructions. RNA purity was quantified using a NanoDrop 2000 spectrophotometer (Thermo Fisher Scientific, New York, NY, United States). Poly(A)-tailed RNA prepared using magnetic oligo (dT) beads was broken into short fragments using a fragment buffer and was then reverse transcribed to synthesize first-strand complementary DNA (cDNA) with a random primer. DNA polymerase I was then mixed with RNase H, deoxyribonucleotide triphosphate (dNTP), and the buffer solution to synthesize the complementary strand. The libraries were constructed using the Illumina methods and protocols, following the manufacturer’s instructions. The insert size and concentration of the cDNA library were both checked and quantified by an Agilent Bioanalyzer 2100 (Agilent Technologies, Inc., Santa Clara, CA) and Qubit^®^ RNA Assay Kit (Life Technologies, CA, United States), respectively. RNA-seq was carried out using an Illumina HiSeq 2500 instrument (Illumina, San Diego, CA, United States). To obtain clean reads and ensure the quality of information analysis, the raw reads were filtered by removing the adapter sequences, empty reads, unknown nucleotides (ratio ≥ 10%), and low-quality reads with a basic mass value of *Q* ≤ 20, which accounted for more than 50% of the whole read length. The clean read assembly was performed according to a previous report (Grabherr et al., 2011). The paired-end clean reads were mapped to the *B*. *mori* genome using the software package TopHat2 (version 2.0.12) (Kim et al., 2013). The genome sequences and annotation file were downloaded from SilkDB. The RNA-seq reads were aligned and then used to construct transcripts with Cufflinks (version 2.1.1) (Trapnell et al., 2012). HTSeq (version 0.6.1) was used to count the reads mapped to each gene to quantify the gene expression levels (Anders et al., 2015). The fragments per kilobase of transcript per million mapped reads (FPKM) of each gene were then calculated based on the length of the gene and read count mapped to a given gene. Genes with a FPKM ≥ 1.0 were identified as “expressed.” A ratio (log_2_ fold change) between the infected and control groups of ≥ 1.5 was identified as the determinant of the DEGs. The raw data have been submitted to the Gene Expression Omnibus (GEO) database with the accession number GSE167875. The functional annotation of DEGs was performed using the Gene Ontology (GO) assignments (Gotz et al., 2008) and Kyoto Encyclopedia of Genes and Genomes (KEGG) pathway enrichments (Kanehisa et al., 2012).

### Tissue Expression Patterns of DEGs

The PGs are important endocrine organs that are significantly different from other tissues in both their morphology and function. In silkworms, the day-3 fifth instar is the boundary for the whole larval development stage (Xu et al., 2019). To analyze the tissue expression patterns of the identified DEGs in the PGs, the PGs, head, integument, midgut, fat body, hemocyte, ovary, testis, Malpighian tubule, trachea, anterior silk gland (ASG), median silk gland (MSG), and posterior silk gland (PSG) of day-3 fifth instar larvae were collected. We detected the expression patterns in the multiple tissues of day-3 fifth instar larvae. Total RNA was extracted using the TRIzol reagent (Invitrogen, Carlsbad, CA, United States). Total RNA concentrations were quantified, and single-stranded cDNAs were synthesized. The *BmGAPDH* gene was used as an intrinsic control.

### RT-qPCR Analysis

The genes selected according to the RNA-seq results were compared by RT-qPCR. Total RNA was extracted from the PGs samples of the infected and control groups at 24, 48, and 72 h. The first-strand cDNA was synthesized using the PrimeScript Reverse Transcriptase kit (TaKaRa, Dalian, China) according to the manufacturer’s instructions. RT-qPCR was performed as previously described (Xu et al., 2019). The *BmGAPDH* gene was used as an intrinsic control (Guo et al., 2016).

## Results

### Statistics of Precocious Maturation of Silkworm After BmNPV Infection

**FIGURE 1:**
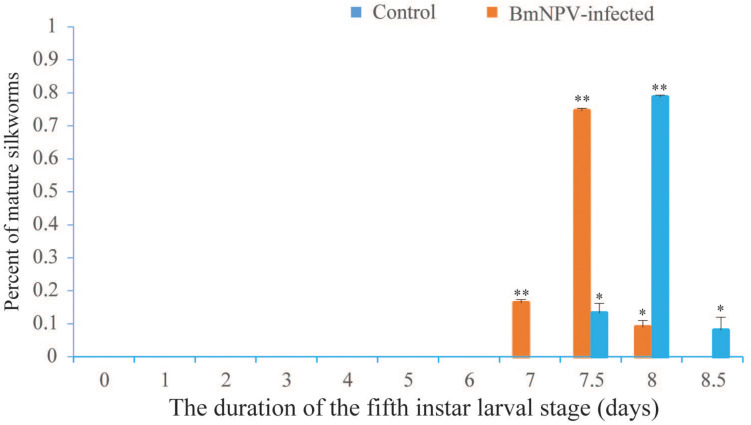
Statistics of the duration of the fifth instar and precocious maturation for day-4 fifth instar silkworm larvae infected with BmNPV. Values represent means ± SDs of three independent investigations. Significant differences are indicated by (*p < 0.05) or (**p < 0.01).

The point when the proportion of mature silkworms was >5% was considered as the first gate, the point when the majority of larvae started maturing was considered as the second gate, and the point when all larvae had reached maturity (100%) was considered as the third gate. The duration of the fifth instar larval stage of the *B. mori* F_50_ strain was almost 8.5 days (Figure 1). The day-4 fifth instar larvae infected with BmNPV matured early. The times that both the first- and second-gate mature silkworms appeared in the infected groups were 12 h earlier than in the control groups (Figure 1). The weights of mature silkworms in the infected groups were significantly decreased when compared with the control groups (Supplementary Table 2). The spinning process was normal, and there was no difference between the infected groups and the control groups. Approximately half of the larvae in the infected groups died during the larval–pupal stage. Compared with the control groups, the cocoon sizes and the weights of the pupae (female and male) were observably reduced in the BmNPV-infected groups (Supplementary Table 2). The fifth instar larvae underwent precocious maturation after infection with BmNPV. Moreover, the day-5 fifth instar larvae were fed with 8,000 mg/L cholesterol and 7dC (via supplemented leaves). The results of feeding with cholesterol and 7dC were also shown to induce precocious maturation when compared to the control, where a certain number of larvae exhibited an anal prolapse in each group fed with cholesterol and 7dC (Supplementary Table 3).

### Assay of Ecdysteroid Titers and RT-PCR Analysis of Viral DNA After BmNPV Infection

**FIGURE 2:**
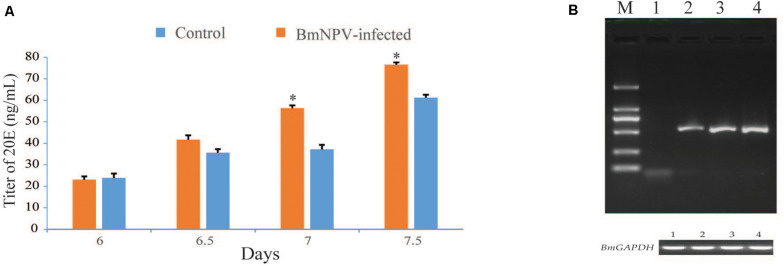
Assay of ecdysteroid titers in hemolymph and examination of virus genomic DNA copies in the prothoracic glands (PGs). **(A)** ELISA analysis was repeated three times for each set of protein samples. Values represent the means ± SDs of three independent determinations. Significant difference is indicated by (*p < 0.05). **(B)** The expression of the BmNPV *GP41* gene was detected in the PGs of the BmNPV-infected silkworm larvae at 24, 48, and 72 h, as well as the control larvae at 48 h. M: DL2000 DNA marker; numbers 1–4 indicate the control group at 48 h and the BmNPV-infected groups at 24, 48, and 72 h, respectively.

Based on the findings of precocious maturation of silkworms after BmNPV infection, the molting and metamorphosis of silkworm requires the presence of 20-hydroxyecdysone (20E). We speculated that the BmNPV infection would have some influences on the ecdysone titer. Thus, the titers of ecdysone in infected and control larvae were determined by ELISA. The results indicated that the ecdysone titers were significantly higher in infected larvae than in the control larvae (Figure 2A). Meanwhile, RT-PCR was used to analyze the virus genomic DNA copies in the PGs at 24, 48, and 72 h after BmNPV infection. The expression of the *BmNPV GP41* gene was detected in the PGs from 24 to 72 hpi (Figure 2B). The expression of the *GP41* gene was not detected in the PGs from the uninfected larvae at 48 h (Figure 2B). These results were useful for selecting the time point for the RNA-seq experiments.

### General Information of RNA-Seq and DEGs

The square of the Pearson correlation coefficient (R^2^) between the four samples was > 0.938 (Supplementary Figure 3). The Q20 values for the clean reads (for each group) were above 95% (Supplementary Table 4). The percentage of clean sequences located on the genome was > 80%. These results indicated that the transcriptome data were assembled with high quality and can be used for further research. The number of expressed genes was 10,152 in the control groups and 10,404 in the BmNPV-infected groups (Supplementary Table 5). In total, seven upregulated and eight downregulated DEGs were screened out (Table 1 and Supplementary Table 6). The functions of the 15 DEGs were primarily located in the binding proteins of nucleic acids, ions, and proteins, and *BmTrypsin-1* had a serine-type endopeptidase activity (Table 1). The DEGs were then annotated by GO analysis to determine their involvement in biological processes, molecular functions, and cellular components (Supplementary Figure 4). The upregulated expression genes were related to biological processes that were mainly focused on metabolic and biological processes (Supplementary Figure 4 and Supplementary Table 7). The downregulated expression genes were focused on biological and metabolic processes and respond to stress factors and stimuli in biological processes (Supplementary Figure 4 and Supplementary Table 7). Regarding the cellular components, only the downregulated expression genes were involved in the membrane and integral components of the membrane, and the upregulated expression genes were not enriched (Supplementary Figure 4 and Supplementary Table 7). Within the molecular function, the upregulated expression genes were primarily located in the catalytic activity, and the down regulated expression genes were involved in protein binding, hydrolase activity, and catalytic activity (Supplementary Figure 4 and Supplementary Table 7). There were some differences in the GO functional annotations between the upregulated and downregulated genes (Supplementary Figure 4). The KEGG pathway enrichment analysis of the identified DEGs showed that the enriched genes were mainly involved in pathways, including metabolic pathways, propanoate metabolism, beta-alanine metabolism, drug metabolism with other enzymes, pyrimidine metabolism, and pantothenate and coenzyme A (CoA) biosynthesis (Table 2 and Supplementary Table 8). The KEGG pathway enrichment analysis of *BmACSS3* revealed that acyl-coenzyme A (AcCoA) synthase activity and *BmACSS3* could be involved in the acyl-CoA to cholesterogenesis pathways.

**TABLE 1:** List of the differentially expressed genes in silkworm prothoracic glands with a 1.5-fold change after BmNPV infection.

	**Gene name**	**Description**	**log_2_ fold change**	**Function**	**ID in silkDB**
**Upregulated**					
1	*BmNGDN*	Eukaryotic translation initiation factor 4E binding protein	1.92	EIF4E binding protein	BGIBMGA003191
2	*BmGag-p*	Gag-pol polyprotein	1.59	Retrotransposon protein	BGIBMGA004024
3	*BmNord*	Neuron-derived neurotrophic factor	2.78	N/A	BGIBMGA008935
4	*BmTrypsin-1*	Trypsin-1 serine protease	2.32	Serine-type endopeptidase activity	BGIBMGA008938
5	*BmACSS3*	Acyl-CoA synthetase short-chain family member 3	1.90	AMP-binging enzyme	BGIBMGA010070
6	*BmJing*	Zinc finger protein jing	3.10	Nucleic acid binding	Novel00232
7	*BmMar1*	Mariner transposon Bmmar1 transposase gene	2.35	DNA binding	Novel00602
**Downregulated**					
8	*BmPyd3*	Carbon–nitrogen hydrolase protein	−2.39	Carbon-nitrogen hydrolase	BGIBMGA001595
9	*BmTitin*	Muscle proteins	−2.82	Protein binding	BGIBMGA002033
10	*BmUnc-89*	Muscle M-line assembly protein unc-89	−2.93	Heterocyclic compound binding	BGIBMGA002034
11	*BmIGc2*	Immunoglobulin C-2 Type	−3.89	Hexosaminidase activity	BGIBMGA004546
12	*BmAryl*	Arylphorin subunit alpha	−1.51	N/A	BGIBMGA008860
13	*BmTitin2*	Muscle proteins	−4.06	protein binding	Novel00168
14	*BmKettin*	Muscle proteins	−5.98	Protein binding	Novel00554
15	*Novel01674*	Uncharacterized protein LOC105842185	−3.84	Ion binding	Novel01674

**TABLE 2:** The Kyoto Encyclopedia of Genes and Genomes (KEGG) pathway enrichment analysis.

**Number**	**Map_Name**	**KEGG_ID**	**Gene_ID**	**Definition**	**log_2_ fold change**
1	Metabolic pathways	bmor:100329149|	BGIBMGA001595	Beta-ureidopropionase	−2.39
		bmor:101743386|	BGIBMGA010070	Fatty acid CoA ligase	1.90
2	Propanoate metabolism	bmor:100329149|	BGIBMGA001595	Beta-alanine synthase	−2.39
3	Beta-alanine metabolism	bmor:101743386|	BGIBMGA010070	Acyl-CoA synthetase	1.90
4	Drug metabolism with other enzymes	bmor:100329149|	BGIBMGA001595	Cyanide hydratase	−2.39
5	Pyrimidine metabolism	bmor:100329149|	BGIBMGA001595	Beta-alanine synthase	−2.39
6	Pantothenate and CoA biosynthesis	bmor:100329149|	BGIBMGA001595	Pantetheine hydrolase	−2.39

### Spatial Expression Patterns of the Identified DEGs

**FIGURE 3:**
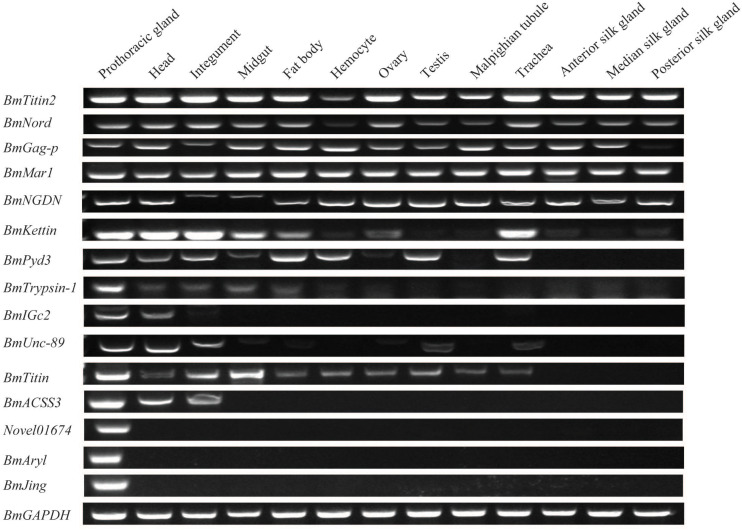
Expression patterns of the differentially expressed genes (DEGs) in multiple tissues of day-3 fifth instar silkworm larvae. RT-PCR was performed, and the *BmGAPDH* gene was used as the internal control.

We investigated the spatial expression patterns of the identified DEGs in multiple tissues of the PGs, head, integument, midgut, fat body, hemocyte, ovary, testis, Malpighian tubule, trachea, ASG, MSG, and PSG of day-3 fifth instar larvae. Such findings can further our understanding of the PGs and elucidate the expression characteristics of DEGs. Expression signals of all of the 15 DEGs were detected in the PGs (Figure 3). The genes of *Novel01674*, *BmJing*, and *BmAryl* were expressed only in PGs (Figure 3). *BmACSS3* was expressed only in the PGs, head, and integument (Figure 3). The other 11 genes were expressed in multiple tissues (Figure 3).

### Expression Analysis of the Identified DEGs

**FIGURE 4:**
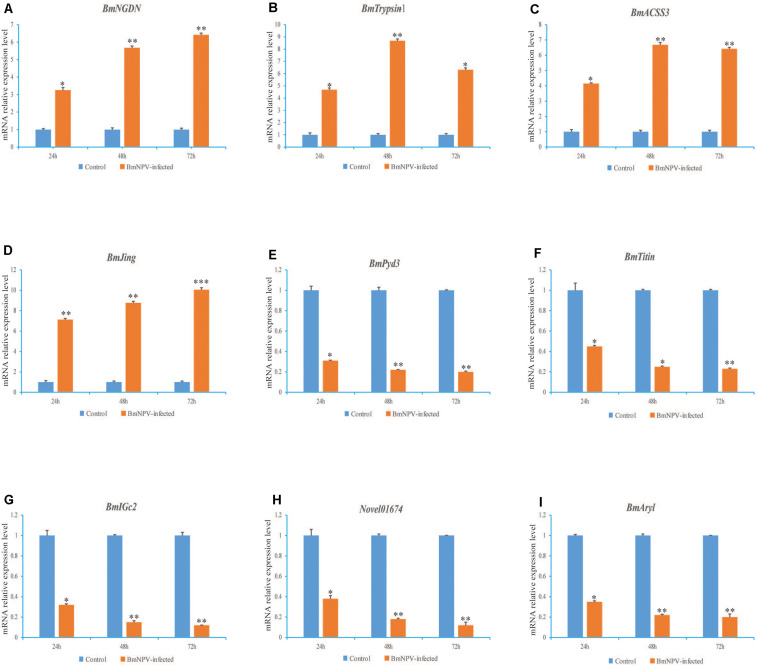
Analysis of the nine randomly selected genes of the differentially expressed genes (DEGs) in the prothoracic glands (PGs) of BmNPV-infected silkworm larvae. The expression levels of *BmNGDN* **(A)**, *BmTrypsin-1* **(B)**, *BmACSS3* **(C)**, *BmJing* **(D)** were upregulated and the expression levels of *BmPyd3* **(E)**, *BmTitin* **(F)**, *BmIGc2* **(G)**, *Novel01674* **(H)**, *BmAryl* **(I)** were downregulated from 24 to 72 h after BmNPV infection, respectively. Here, *BmGAPDH* was used as the internal control. The experiments were repeated three times. Values represent the means ± SDs of three independent experiments. Significant differences are indicated by (*p < 0.05) or (**p < 0.01).

We used RT-qPCR to investigate the relative expression levels of nine randomly selected genes of the DEGs in the PGs of BmNPV-infected larvae at 24, 48, and 72 h. The expression levels of *BmNGDN*, *BmTrypsin-1*, *BmACSS3*, and *BmJing* were upregulated in the transcriptome data, i.e., their transcripts were significantly increased from 24 to 72 h after BmNPV infection (Figures 4A–D). Meanwhile, the expression levels of *BmPyd3*, *BmTitin*, *BmIGc2*, *Novel01674*, and *BmAryl* were downregulated in the transcriptome data, i.e., their transcripts were significantly decreased from 24 to 72 h after BmNPV infection (Figures 4E–I). The changes in the transcription of the nine genes were generally consistent with the transcriptome data.

## Discussion

The mulberry silkworm is one of the best models to study insect physiology and biochemistry, especially to better understand the relationship between induction factors (external and internal) and development. In general, the larvae of tetramolter silkworms proceed through five instars and undergo molting between each instar. The last instar larva completes the larval–pupal transition. The developmental speed of silkworm larvae has been shown to be regular and constant within the same silkworm strain and when maintained under the same rearing conditions. The day-3 fifth instar larval stage is considered to represent the boundary for the larval stage.

In this study, the day-4 fifth instar larvae infected with BmNPV (using 2.0 × 10^6^ OB/larva) matured early, and the ecdysone titer in infected larvae was significantly higher than that of the control larvae. In addition, BmNPV infection (using 2.0 × 10^7^ OB/larva) also caused larvae precocious maturation (only at the first gate), followed by illness and death (data not shown). Meanwhile, day-5 fifth instar larvae fed with cholesterol and 7dC also exhibited precocious maturation. Cholesterol and 7dC supplementation in the latter half of the fifth instar shortened the fifth instar period. In contrast, cholesterol and 7dC supplementation in the first half of the fifth instar (days 1–3) prolonged the fifth instar period, but no results were observed for BmNPV infection in the first half of the fifth instar because the larvae died. Briefly, the prothoracicotropic hormone (PTTH) secreted by the brain stimulates the PGs to release ecdysteroid, which in turn induces larval or metamorphic ecdysis depending on the presence of juvenile hormone (JH) secreted by the corpora allata. Throughout the latter half of the fifth instar, the first step of the ecdysteroid titer increased in a stepwise manner, the second step of the ecdysteroid titer showed a small increase (which led to the silkworms wandering) followed by a plateau, and the third step of the ecdysteroid titer showed an initially gradual but then steep increase to reach a peak 1 day later (Mizoguchi et al., 2001).

Insights from host studies reveal that baculoviruses manipulate host behavior to enhance transmission to new victims. For example, baculoviruses enable infected larvae to continue to seek foliage and prolong insect feeding after infection, thus resulting in an increased OBs production (O’Reilly and Miller, 1989). The *egt* gene of NPV, expressed immediately, encodes an enzyme that inactivates the molting hormone 20E by transferring a sugar moiety from a nucleotide sugar donor to a hydroxyl group on 20E (Hoover et al., 2011). The ecdysone blood level is reduced by up to 90% in silkworms as a result of the transgenic expression of the *egt* gene of BmNPV and because *egt* expression in *egt*-transgenic silkworms prolongs the duration of the larval and pupal stages resulting in the arresting of the pupal-to-adult metamorphosis (Zhang et al., 2012). Interestingly, in the present study, silkworm larvae infected with BmNPV in the latter half of the fifth instar showed precocious molting and metamorphosis and a higher level of hemolymph ecdysone titer. The *egt* gene of BmNPV is dispensable for normal virus production (Katsuma et al., 2008). The fast-killing phenotype is observed in the three *egt*-mutated BmNPVs only when the infection process progresses through silkworm larval–larval transition, but under infection in the middle stages of the fifth instar, the slow-killing phenotype is observed than that of the wild-type virus-infected larvae (Katsuma and Shimada, 2015). In particular, in the gluttonous stage, silkworms synthesize an enormous amount of silk proteins in the silk gland (Xu et al., 2019), and silk proteins are dispensable for normal silkworm development. A certain amount of silk proteins can remain in the body (incomplete spinning) that can lead to an incomplete larval–pupal transition. Silkworm and BmNPV interactions are largely dependent on the developmental stage of the host larvae infected by the virus. In addition, the overproduction of silkworm PTTH induces higher than normal levels of hemolymph ecdysteroids, which have been found to inhibit the pathogenicity of the virus, but did not have any observable effects on the development of infected *Spodoptera frugiperda* larvae (O’Reilly et al., 1995). Moreover, insect innate immunity can be activated by 20E and 20E, which induce antimicrobial peptide (AMP) gene expression and thus act as immune activators (Dimarcq et al., 1997; Roxstrom-Lindquist et al., 2005; Flatt et al., 2008). The ecdysteroid titer showed a small increase followed by a plateau that occurred 1 day before the silkworms started wandering. Thereafter, the titer increased gradually and then steeply to reach a peak (where the majority of silkworms had started wandering) the following day (Mizoguchi et al., 2001). Therefore, we speculated that BmNPV infection in the latter half of the fifth instar of silkworm larvae induced precocious molting and metamorphosis and a higher level of hemolymph ecdysone titer, which would enable infected larvae to complete their larval–pupal transition.

Briefly, PGs are an important endocrine organ with characteristically cholesterol-rich tissue as the main site of synthetic ecdysteroids (Igarashi et al., 2018). In this study, the RT-PCR results confirmed that the silkworm PGs were infected by BmNPV through oral inoculation. Seven upregulated and eight downregulated DEGs were identified from silkworm PGs sequenced by RNA-seq 48 h after BmNPV infection. The RT-qPCR results of the DEGs in the PGs of BmNPV-infected larvae at 24, 48, and 72 h were generally consistent with the transcriptome data. The classifications of the 15 DEGs were primarily located in binding activity of nucleic acids, ions, and proteins that were mainly involved in the metabolic processes and pathways. The spatial expression profiles of *Novel01674*, *BmAryl*, and *BmJing* indicated that they were specifically expressed in the silkworm PGs. The KEGG pathway enrichment analysis of *BmACSS3* (BGIBMGA010070) revealed that acyl-coenzyme A (AcCoA) synthase activity and *BmACSS3* could be involved in the acyl-CoA to cholesterogenesis pathways. The acyl-coA synthetase catalyzes fatty acids to form a thioester with CoA, which is a common initial step of all fatty acid metabolic processes (Watkins, 1997). Fatty acids are the building blocks of many lipids, including triacylglycerol and cholesteryl esters. RNAi-mediated knockdown of the acyl-coenzyme A synthetase gene prolongs and extends the maximum lifespan (Eisenberg et al., 2014). Trypsin-1 serine protease (*BmTrypsin-1*) had serine-type endopeptidase activity. SPs play crucial roles in insect development and innate immunity. RNAi-mediated silencing of SPs results in severe molting defects, specifically by reducing the expression of genes in the 20E synthesis and signaling pathway, and increases larval sensitivity to bacteria (Broehan et al., 2010; Yang et al., 2019). In silkworm PGs, the transcripts of *BmTrypsin-1* and *BmACSS3* were significantly increased from 24 to 72 h after BmNPV infection. *BmTrypsin-1* and *BmACSS3* may be involved in the maturation process in the latter half of the fifth instar of silkworm larvae.

## Data Availability Statement

The datasets presented in this study can be found in online repositories. The names of the repository/repositories and accession number(s) can be found in the article/Supplementary Material.

## Author Contributions

P-ZX performed the experiment and wrote the manuscript. M-RZ performed the literature review and analyzed the data. X-YW prepared the illustrations and collected the data. Y-CW suggested important research points. All authors have read and approved the final version of the manuscript.

## Conflict of Interest

The authors declare that the research was conducted in the absence of any commercial or financial relationships that could be construed as a potential conflict of interest.

## Supplementary Material

Supplementary Figure 1 View of the prothoracic glands (PGs) entwined in the tracheal bush of the first spiracle.

Supplementary Figure 2 View of the prothoracic glands (PGs) from a fifth instar silkworm larva.

Supplementary Figure 3 Analysis of the correlation of RNA-seq data. **(A)** Diagram of the correlation coefficients between samples. **(B)** Correlation between C1 and C2. **(C)** Correlation between C1 and N1. **(D)** Correlation between C1 and N2. **(E)** Correlation between C2 and N1. **(F)** Correlation between C2 and N2. **(G)** Correlation between N1 and N2. C1 and C2 indicate the two independent biological experiments of transcriptome sequencing of prothoracic glands (PGs) in the control groups, respectively. N1 and N2 indicate the two independent biological experiments of transcriptome sequencing of PGs in the control groups, respectively.

Supplementary Figure 4 GO enrichment analysis of the differentially expressed genes (DEGs). Genes were annotated by the biological process, cellular component, and molecular function.

Supplementary Table 1 Primers of genes for RT-PCR and RT-qPCR.

Supplementary Table 2 The weight of mature silkworms and pupae in BmNPV-infected and control groups.

Supplementary Table 3 The results of feeding experiment with cholesterol, and 7-dehydrocholesterol (7dC), using water as control.

Supplementary Table 4 Summary statistics of prothoracic gland of *Bombyx mori* genes based on transcriptome data.

Supplementary Table 5 A list of genes with FPKM ≥ 1.0 between infected and control groups.

Supplementary Table 6 A list of differential expression genes between infected and control groups.

Supplementary Table 7 Infected vs. control DEG_GO_enrichment result.

Supplementary Table 8 Infected vs. control all DEG_KEGG_pathway_enrichment result.
