# Supplementary figures and images for: Precocious Metamorphosis of Silkworm Larvae Infected by BmNPV in the Latter Half of the Fifth Instar
Source: Front Physiol. 2021 May 10;12:650972. doi: 10.3389/fphys.2021.650972 (PMC8141865; doi:10.3389/fphys.2021.650972)

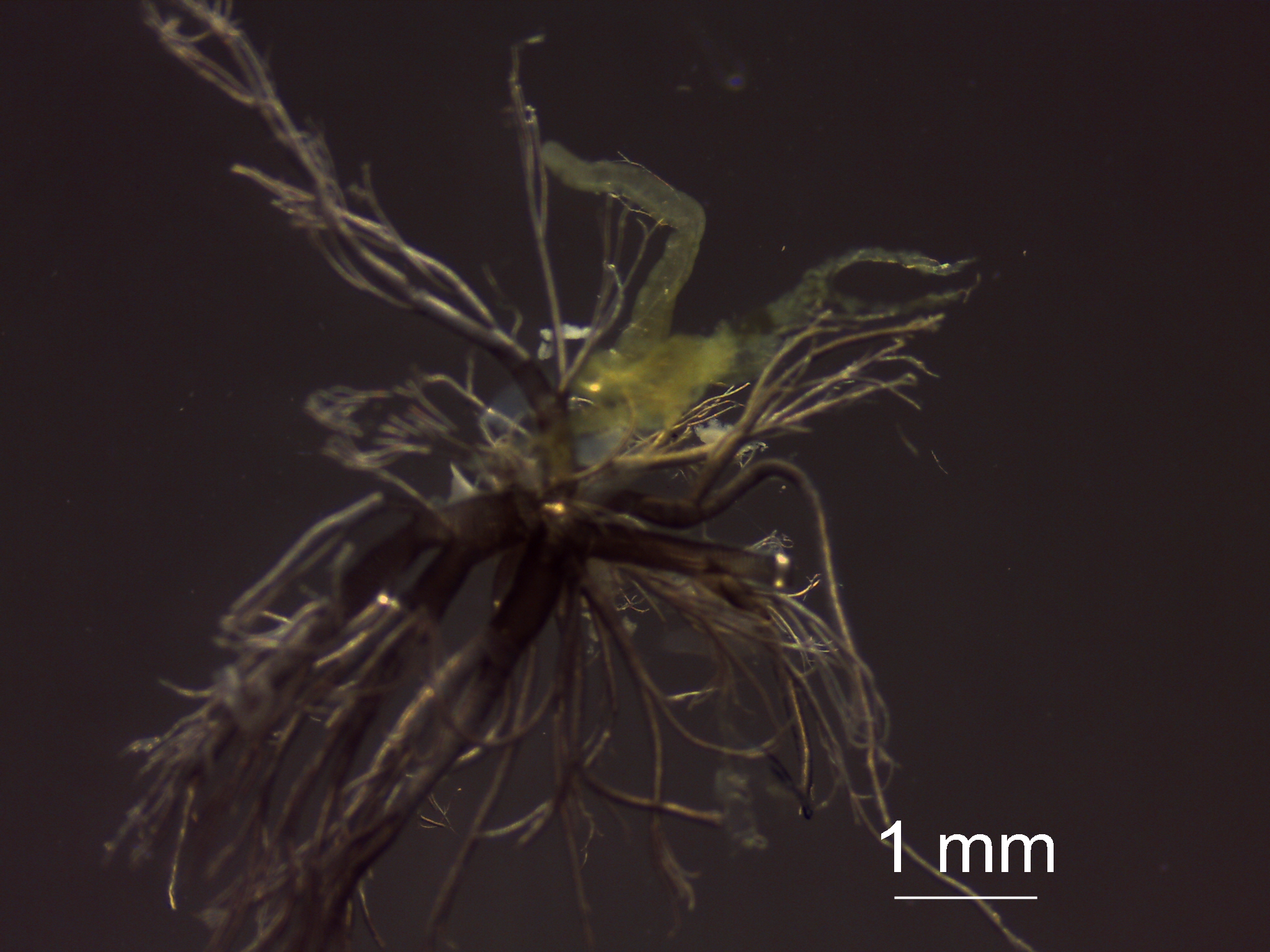

Supplement: Supplementary Figure 1 — View of the prothoracic glands (PGs) entwined in the tracheal bush of the first spiracle. [file Image_1.tif]

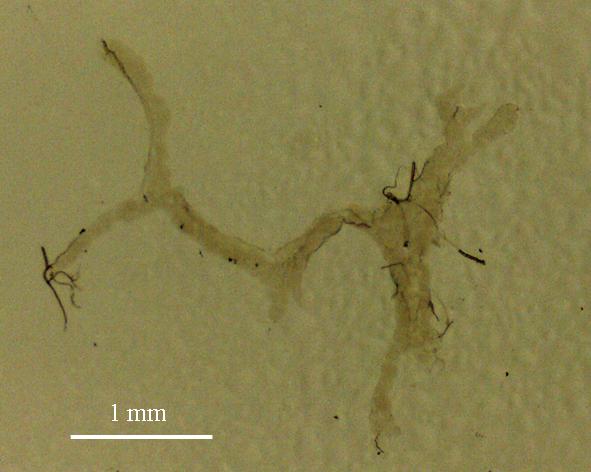

Supplement: Supplementary Figure 2 — View of the prothoracic glands (PGs) from a fifth instar silkworm larva. [file Image_2.jpeg]

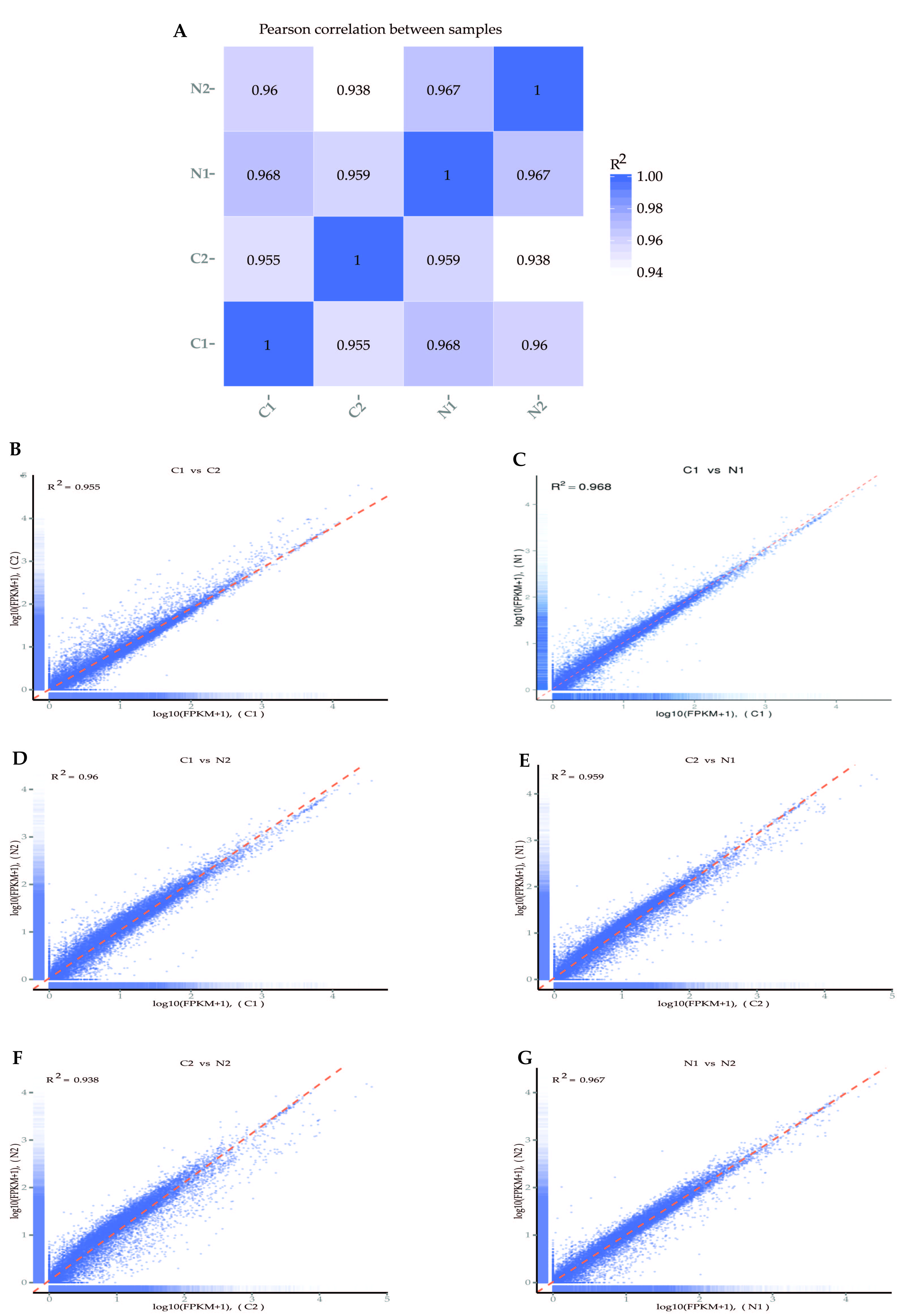

Supplement: Supplementary Figure 3 — Analysis of the correlation of RNA-seq data. (A) Diagram of the correlation coefficients between samples. (B) Correlation between C1 and C2. (C) Correlation between C1 and N1. (D) Correlation between C1 and N2. (E) Correlation between C2 and N1. (F) Correlation between C2 and N2. (G) Correlation between N1 and N2. C1 and C2 indicate the two independent biological experiments of transcriptome sequencing of prothoracic glands (PGs) in the control groups, respectively. N1 and N2 indicate the two independent biological experiments of transcriptome sequencing of PGs in the control groups, respectively. [file Image_3.jpeg]

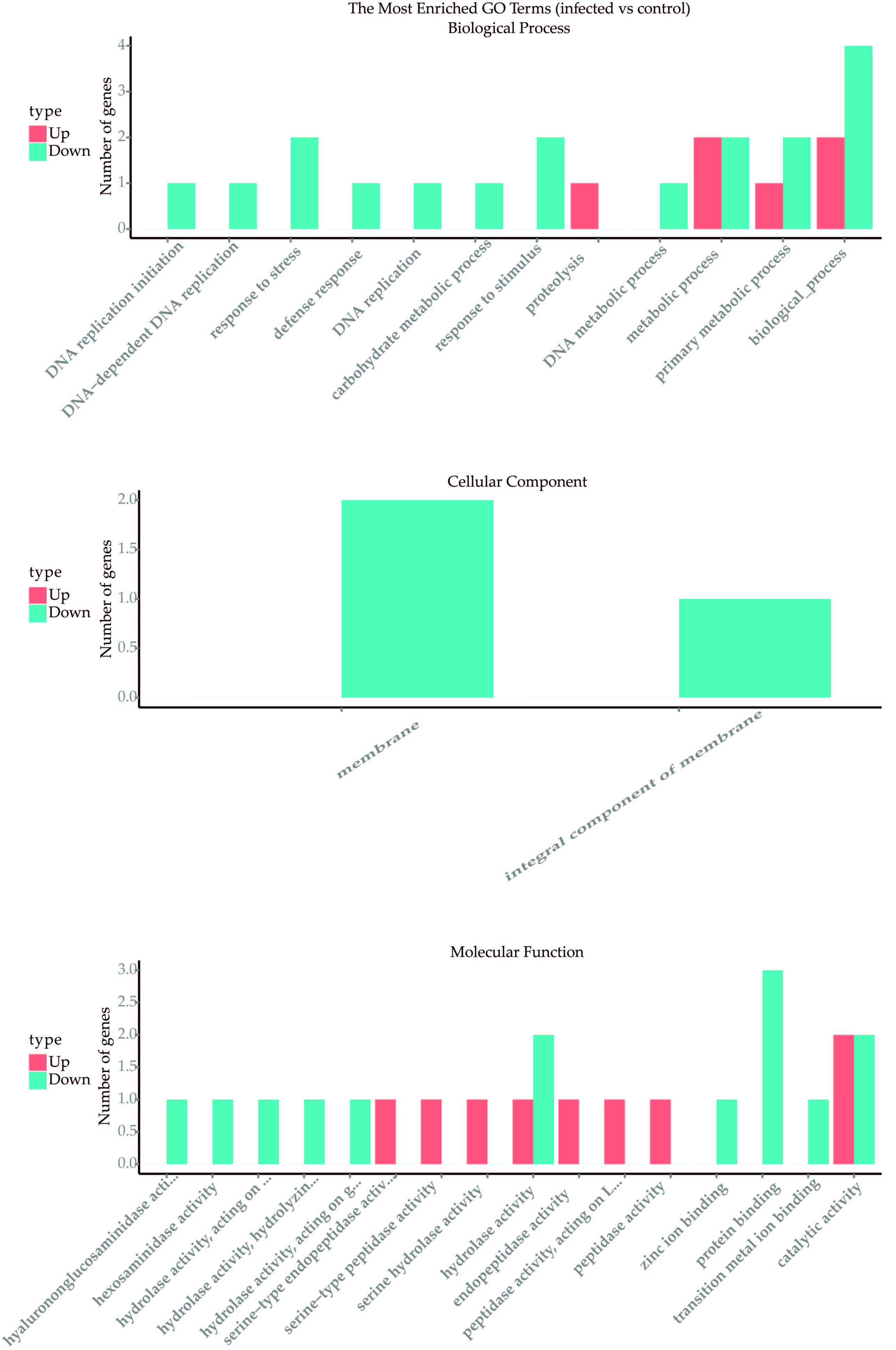

Supplement: Supplementary Figure 4 — GO enrichment analysis of the differentially expressed genes (DEGs). Genes were annotated by the biological process, cellular component, and molecular function. [file Image_4.jpeg]
